# *M. tuberculosis* Peptide based Point-of care test for TB

**DOI:** 10.1186/1471-2334-14-S3-E27

**Published:** 2014-05-27

**Authors:** Jesus M Gonzalez, Bryan Francis, Sherri T Burda, R Sam Niedbala, Suman Laal

**Affiliations:** 1 TB Biosciences, 205 Webster Street, Bethlehem, PA, USA; 2 Lehigh University, Department of Chemistry, Bethlehem, PA, USA; 3 Veterans Affairs Medical Center, New York Harbor Health Care System, New York, NY, USA; 4 New York University Langone Medical Center, Departments of Pathology and Microbiology, New York, NY, USA

## Background

A rapid, simple and affordable point-of care (POC) test that can be implemented in peripheral health care settings to replace/improve upon microscopy for TB diagnosis or to rule out TB (triage) remains unmet.

## Methods

Immunodominant regions of 3 *Mycobacterium tuberculosis* specific cell–wall proteins that are highly immunogenic in HIV- TB+ and HIV+TB+ patients have been mapped. Antibodies to these regions are absent in subjects with latent TB, BCG vaccination, other pulmonary diseases. To examine the feasibility of devising a peptide based rapid POC test, selected peptides were conjugated to a carrier protein using standard EDC/NHS coupling chemistry, purified by size exclusion; the peptide conjugation confirmed using MALDI MSMS before adsorption onto colloidal gold and striping onto nitrocellulose. The assay parameters were adjusted to determine positive/negative status within 15 minutes via visual or instrumented assessment.

## Results

The prototype POC test was evaluated with sera from ~400 TB patients, non TB subjects from the groups defined in the methods section. The current prototype POC test provides >90% sensitivity and specificity in the above populations.

## Conclusion

WHO recommends against use of all current commercial serological TB tests since they fail to achieve adequate sensitivity and specificity. These results demonstrate that, as for HIV rapid tests, carefully selected immunodominant epitopes of highly antigenic *M. tuberculosis* specific proteins can be the basis of antibody detection based rapid POC TB test. Test optimization to improve accuracy to meet WHO specifications for a POC test to replace microscopy or a triage test to rule-out TB are ongoing.

